# Effects of Smoking and Non-Insulin-Dependent Diabetes Mellitus on Blood Trace Element Levels

**DOI:** 10.7759/cureus.72618

**Published:** 2024-10-29

**Authors:** Ahmed M Ahmed, Awadh S Alsubhi, Turki S Shawosh, Mouath Almuntashiri, Abdullah K Alebire, Faisal F Mohammadi, Abdulmannan M Aman

**Affiliations:** 1 Clinical Laboratory Sciences, College of Applied Medical Sciences, Taibah University, Medina, SAU; 2 Clinical Laboratory Sciences, Taibah University, Medina, SAU; 3 University Medical Center, Taibah University, Medina, SAU

**Keywords:** d&s, os, oxidative stress, smokers, t2dm, trace elements

## Abstract

Background: The connection between oxidative stress and trace elements is linked to various diseases and their development and consequences. This relationship is complex due to the alterations caused by oxidative stress.

Methods: A total of 100 patients with type-2 diabetes mellitus (T2DM) who never smoked, 100 smokers, and 90 diabetes patients who smoked cigarettes (D&S) were compared with 100 healthy subjects. Serum trace elements, glycemic profiles, antioxidants, lipids, and malondialdehyde (MDA) were measured for all participants.

Results: The results showed a high cholesterol level in D&S subjects (p < 0.01). Zinc (Zn), magnesium (Mg), and chromium (Cr) were reduced in T2DM and D&S patients (p < 0.05). Copper (Cu) and Cr were higher in smokers and D&S (p < 0.01). Mg and Zn were correlated with superoxide dismutase (SOD) and glutathione peroxidase (GPx) in the control group (p < 0.05). Zn was inversely correlated with glucose in T2DM (p < 0.05) and with MDA in smokers and D&S (p < 0.01). In addition, Cu and Cr were correlated with MDA in smokers (p < 0.01). Moreover, potassium (Kalium, K) was correlated with glucose in T2DM (p < 0.01).

Conclusion: Decreased Mg and Zn in patients with diabetes indicate that diabetes may contribute to the decrease of these elements, whereas high levels of Cu and Cr have been associated with increased oxidative stress. This suggests that smoking is a major cause of oxidative stress.

## Introduction

Trace elements, also known as micronutrients, are inorganic compounds that occur naturally. They are necessary for a range of physiological and metabolic functions, such as food breakdown, enzyme activation, hormone production, cell growth, and immune defense [1]. Oxidative stress (OS) results from a disrupted balance between the body's antioxidant defense mechanisms and reactive oxygen species (ROS) production. Trace elements such as iron, zinc, and copper are essential for enzyme function and antioxidant defense against OS. Cu and Zn are necessary building blocks of superoxide dismutase (SOD) and vital for signaling molecules and catalytic activity as cofactors [2]. Antioxidants such as glutathione peroxidase (GPx), catalase (CAT), and SOD are the primary defense mechanisms against oxidative damage [3].

It is well documented that OS is more common in older individuals and those with poor habits such as an unhealthy diet or smoking, with diabetes being one of the diseases associated with these factors [4].

Diabetes is known to trigger ROS formation, leading to OS due to advanced glycation end products and NADH oxidation. Obesity leads to the accumulation of fats in tissues and organs, resulting in lipotoxicity, inflammation, and systemic OS due to an excess of adipose tissue [5].

Tobacco smoke, which contains ROS and nitrogen species, may increase inflammation and OS, potentially contributing to the development and progression of cancer [6].

This study aims to analyze how OS and trace elements are related to metabolic disorders and lifestyle choices in selected Saudi people who participate in this study.

## Materials and methods

Subjects

The study population included 100 patients with type-2 diabetes mellitus (T2DM) who do not smoke, 100 cigarette smokers, and 90 patients with T2DM who do smoke (D&S). They were compared to a control group of 100 healthy individuals. Patients were selected from the University Medical Center at Taibah University (Medina, KSA) during the period of July 2022 to May 2024. Patients were selected according to a simple randomized design. The inclusion criteria for the diabetic group comprised HbA1c ≥ 6.5% and fasting blood glucose (FBG) ≥ 7.7 mmol/L. People suffering from acute diseases or using vitamin/mineral supplements were excluded.

Ethical approval

The study was approved by Taibah University's Applied Medical Sciences research ethics committee (no: CLS 2021019), following the Helsinki Declaration of 1964 and all amendments. Written informed consent was obtained from all participants.

Anthropometrics

Body mass index (BMI) and waist circumference (WC) were determined as previously mentioned [7]. BMI was classified as follows: normal weight (18.5-24.9 kg/m^2^), overweight (25-29.9 kg/m^2^), and obese (≥30.0 kg/m^2^).

Laboratory parameters

Fasting blood samples were collected from the participants. Glucose, lipids, and electrolytes were enzymatically measured using the Dimension® EXL™ 200 Integrated Chemistry Analyzer (Siemens, Erlangen, Germany). HbA1C levels were determined using the D-10™ Hemoglobin Analyzer from Bio-Rad (NycoCard, Hercules, California, USA). Trace elements (Zn, Mg, Mn, Cu, Fe, Ca, Cr, and Se) were measured using the Atomic Absorption Spectrophotometer WFX- 320 from Beijing Rayleigh Analytical Instrument Corporation (BRAIC, Beijing, China). Antioxidants and GPx levels were measured according to changes in NADPH, which was spectrophotometrically read at 340 nm. SOD was determined using the principle of nitro blue tetrazolium reduction rate. Malondialdehyde (MDA) was measured using thiobarbituric acid reactive substances (TBARS) [8].

Statistical analysis

Data analysis was conducted using IBM SPSS Statistics for Windows, Version 19 (Released 2010; IBM Corp., Armonk, New York). Continuous variables were compared through an unpaired Student's t-test, Pearson correlation, ANOVA for multiple groups, and chi-square for categorical variables, with p < 0.05 denoting significant variations. The GraphPad Prism Software (San Diego, CA, USA) was used for figure design.

## Results

**Figure 1:**
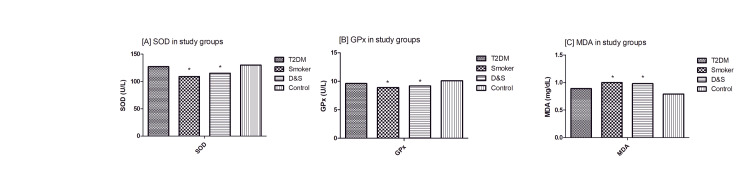
Comparison of the mean level of antioxidants and MDA between the study groups *Statistically significant compared to control (p < 0.01) T2DM: type 2 diabetes mellitus; D&S: diabetes and smoking; MDA: malondialdehyde; SOD: superoxide dismutase; GPx: glutathione peroxidase

Figure 1 represents the comparison of antioxidants and MDA levels among participants. SOD and GPx levels were found to be lower in the smokers and D&S groups than that in the control group (p < 0.01). Additionally, MDA levels were higher in the smokers and D&S groups than that in the control group (p < 0.01).

Table 1 contains the demographic and clinical data of the participants. Ages and genders were matched between control and clinical populations. Glucose and HbA1c levels were higher in the T2DM and D&S groups than that in the smokers and control groups (p < 0.001). Additionally, TC was higher in the D&S group than that in the control group (p < 0.01).

**Table 1:** General characteristics and biochemical profiles of the study population *Statistically significant at p < 0.05 **Statistically significant at p < 0.01 ^a^High compared to the smoker and control groups (p < 0.01) ^b^High compared to the control group (p < 0.01) T2DM: type 2 diabetes mellitus; D&S: diabetes and smoking; No.: number; BMI: body mass index; WC: waist circumference; FBG: fasting blood glucose; HbA1C: hemoglobin A1C; TC: total cholesterol; TG: triglyceride; LDL: low-density lipoprotein; HDL: high-density lipoprotein

Parameters	T2DM	Smoker	D&S	Control	P-value
No.	100	100	90	100	-
Age (years)	47±15.7	42±18.1	43±16.1	46±13.5	0.08
Gender (male, female)	61, 39	87, 13	79, 11	60, 40	-
BMI (kg/cm^2^)	23.8±9.8	23.2±8.1	23.8±11.5	22.2±9.7	0.62
WC (cm)	84.9±20.5	82.1±18.5	86.1±21.1	81.9±20.9	0.38
FBG (mmol/L)	8.5±3.3^a^	4.6±1.1	8.6±3.1^a^	4.5±1.2	<0.001^**^
HbA1c (%)	7.8±2.9^a^	5.6±1.3	7.9±3.4^a^	5±1.1	<0.001^**^
TC (mmol/L)	3.85±2.6	4.07±3.2	4.7±2.9^b^	3.43±1.5	0.01^*^
TG (mmol/L)	1.62±1.45	1.59±1.3	1.76±1.6	1.36±1.2	0.25
LDL (mmol/L)	2.56±1.6	2.59±1.9	2.69±2	2.3±1.2	0.42
HDL (mmol/L)	1.17±0.4	1.19±0.2	1.11±0.6	1.23±0.2	0.18

Table 2 shows the comparison of trace element levels across the study groups. Mg, Zn, Cu, and Cr exhibited significant differences (p < 0.05). Additionally, Mg and Zn were lower in the T2DM and D&S groups than that in the control group (p < 0.01). Cu was also lower in the control and T2DM groups than that in the D&S and smokers groups (p < 0.01).

**Table 2:** Trace and macro elements in the study groups *Statistically significant at p < 0.05 **Statistically significant at p < 0.01 ^a^High compared to the T2DM and D&S groups (p < 0.01) ^b^High compared to the T2DM and D&S groups (p < 0.05) ^c^High compared to the T2DM and control groups (p < 0.01) ^d^High compared to the T2DM and control groups (p < 0.01) T2DM: type-2 diabetes mellitus; D&S: diabetes and smoking

Trace Elements	T2DM	Smoker	D&S	Control	P-value
Calcium (mmol/L)	2.1±1	2.2±0.4	2.1±0.8	2.4±1.3	0.08
Magnesium (mmol/L)	0.82±0.1	0.83±0.3	0.8±0.1	0.91±0.3^a^	0.004**
Zinc (µmol/L)	15.1±3.2	16.2±6.3	14.9±3.7	16.9±6.1^b^	0.01*
Manganese (µg/L)	0.25±0.01	0.26±0.09	0.25±0.06	0.27±0.03	0.38
Copper (µg/dL)	123±20.2	139±41.5^c^	140±32.7^c^	122±26.7	<0.001**
Sodium (mmol/L)	144.2±22.7	142.6±27.1	143.2±30.3	142.1±26.1	0.95
Potassium (mmol/L)	4.8±2.2	4.3±1.2	4.7±2	4.4±1.1	0.11
Iron (µmol/L)	19.3±16.2	17.2±10.7	18.2±12.1	22.1±13.9	0.06
Chromium (ng/mL)	0.66±0.3	1.3±1^d^	1.1±0.5^d^	0.6±0.1	<0.001**
Selenium (µg/L)	144.3±45.5	133.2±39.2	136.2±29.7	142.6±39.2	0.06

Table 3 represents the correlation between trace elements and glucose, antioxidants, and MDA in the different study groups. Mg and Zn were correlated with SOD and GPx in the control group (p < 0.05). Zn was inversely correlated with glucose in the T2DM group (p < 0.05) and with MDA in the smokers and D&S groups (p < 0.01). Additionally, Cu and Cr were correlated with MDA in the smokers and D&S (p < 0.01). Furthermore, K was correlated with glucose in the T2DM group (p < 0.01).

**Table 3:** Correlation between trace elements level across the study groups vs. FBG, MDA, and antioxidants levels *Statistically significant at p < 0.05 **Statistically significant at p < 0.01 T2DM: type-2 diabetes mellitus; D&S: diabetes and smoking; FBG: fasting blood glucose; MDA: malondialdehyde; SOD: superoxide dismutase; GPx: glutathione peroxidase

Trace Elements	T2DM				Smoker				D&S				Control			
	FBG	MDA	SOD	GPx	FBG	MDA	SOD	GPx	FBG	MDA	SOD	GPx	FBG	MDA	SOD	GPx
Ca	0.07	0.04	0.1	0.1	0.05	0.04	0.08	0.04	0.06	0.08	0.02	0.02	0.09	0.09	0.1	0.2
Mg	-0.03	0.03	0.2	0.2	-0.06	0.07	0.05	0.06	-0.06	0.04	0.1	0.2	0.01	0.03	0.4*	0.2*
Zn	-0.3*	-0.1	0.3	0.4	-0.02	-0.6*	0.2	0.1	-0.2	-0.5*	0.2	0.09	-0.02	-0.2	0.3*	0.4*
Mn	0.03	0.1	0.09	0.2	0.02	0.05	0.02	0.09	0.02	0.04	0.02	0.09	0.03	0.01	0.2	0.1
Cu	0.02	0.2	0.1	0.1	0.04	0.6**	0.04	0.05	0.03	0.5**	0.1	0.09	0.06	0.02	0.1	0.1
Na	-0.18	0.09	0.06	0.09	0.09	0.19	0.05	0.03	0.1	0.2	0.07	0.07	0.1	0.04	0.09	0.06
K	0.6**	0.06	0.02	0.09	0.3	0.16	0.06	0.05	0.3	0.12	0.05	0.06	0.3	0.07	0.05	0.07
Fe	0.02	0.08	0.1	0.1	0.1	0.07	0.08	0.08	0.2	0.09	0.1	0.1	0.09	0.05	0.2	0.2
Cr	0.04	0.1	0.07	0.06	0.07	0.7**	-0.06	-0.07	0.08	0.6**	-0.1	-0.09	0.01	0.03	0.1	0.1
Se	0.02	0.09	0.03	0.01	0.06	0.22	0.01	0.02	0.03	0.2	0.06	0.03	0.01	0.06	0.06	0.09

## Discussion

The present research analyzed and compared trace element levels between healthy individuals, those with T2DM, and smokers. Zinc, magnesium, and chromium levels were lower in individuals with T2DM, while copper and chromium levels were found to be elevated in smokers. Moreover, copper levels were elevated in individuals with T2DM and those who smoke compared to healthy controls.

Zn and Mg ions are reduced in T2DM patients and obese individuals, which is in accordance with a previous study [9]. This could be attributed to the fact that they are essential for glucose and lipid metabolism, insulin production, and antioxidant defense system performance [10]. Zinc promotes antioxidant enzyme production and prevents metabolic syndrome by inhibiting proinflammatory cytokines and reducing ROS production. All living organisms require these ions [11].

SOD, a protein with zinc in its cytoplasm, converts receptive species into less dangerous ones, reducing the poisonous quality of ROS that require sufficient zinc levels in cell compartments. Homma et al. [12] demonstrated that Zn loss could be attributed to SOD. It could cause mutant-like conformation and persistent endoplasmic reticulum stress, resulting in Zip-14 transporter induction and protein synthesis inhibition. In the present study, zinc strongly correlated with SOD and GPx enzyme concomitant with an inverse correlation with MDA, which agrees with studies from Japan, Poland, and Greece [12]. The possible correlation between Zn and OS may be due to chronic inflammation leading to glucocorticoid synthesis, which activates metallothionein and Zn transporter genes and ultimately increases Zn absorption by affecting its distribution and plasma levels in adipocytes [13]. Magnesium is crucial for glucose and insulin regulation, protein synthesis, DNA and RNA, mitochondrial function, and calcium homeostasis. ATP production (Mg is bound to nucleotide triphosphates (MgATP)) [14]. A decreased Mg level is linked to cardiovascular mortality [15].

This study found that levels of Mg were lower in T2DM and D&S groups compared to the control group, which is consistent with research conducted in America and India [16,17]. Individuals with low levels of magnesium are at risk of developing conditions such as diabetes and obesity because the heart contains transporters and regulator channels that control magnesium movement, including TRPM7, TRPM7, MagT1, and CNNM2 for magnesium influx and SLC41A1 and SLC41A3 for magnesium efflux. Disruption of channels can disturb magnesium balance, which leads to magnesium-ATP interactions in mitochondrial signaling. This may serve as an explanation for the excessive ROS production and decreased ATP levels observed in magnesium deficiency [16].

Copper is essential for human health as it acts as a cofactor for enzymes and electron providers. It also contributes to energy production, leading to a distortion in the mitochondria of cells that are active in metabolism, such as those found in the liver and pancreas [18,19]. In the current research, smokers showed an increase in Cu levels, which is consistent with findings from studies conducted in Italy and Finland [20,21]. Cigarettes and tobacco have been found to contain copper [21], which can disrupt hormones and increase ceruloplasmin and metallothionein protein production. Copper triggers OS by enhancing the generation of ROS via a Fenton-like reaction, leading to a decrease in glutathione levels [22].

Furthermore, there is a correlation between copper and OS, which is consistent with research findings from China [23,24]. It controls the adipocyte energy utilization process [24] and raises lipid peroxidation levels [25]. Cu was not associated with an increased risk of T2DM, contradicting research from Taiwan and Egypt [18,26] while supporting findings from China [27], Austria [28], and Sudan [9].

Cr levels were found to be higher in smokers and D&S patients, which is consistent with research conducted in Jordan [29]. Chromium plays a crucial role in various metabolic processes in the human body, especially the metabolism of fats and carbohydrates.

Limitations

The study's limitations include its limited generalizability, the lack of adjustment for confounders, such as age, diet, and medications, its cross-sectional nature and inability to infer causality, and the gender imbalance in the groups, which could have affected the results. Incorporating these points into future research would strengthen critical evaluation and provide a more balanced perspective.

## Conclusions

Mg and Zn levels were found to be decreased in the T2DM and D&S groups, which indicates that diabetes may contribute to the decrease of these elements. Higher levels of Cu and Cr in the smokers and D&S groups suggest that smoking is a major cause of elevated levels of these elements. Higher levels of antioxidants Mg and Zn have been observed in healthy individuals, while higher levels of Cu and Cr have been associated with increased OS in smokers; MDA is higher in the smokers and D&S groups.
